# Adapting hip arthroplasty practices during the COVID-19 pandemic: Assessing the impact of outpatient care sudden increase on early complications and clinical outcomes

**DOI:** 10.1051/sicotj/2023037

**Published:** 2024-01-09

**Authors:** Constant Foissey, Tomas Pineda, Elvire Servien, Andreas Fontalis, Cécile Batailler, Sébastien Lustig

**Affiliations:** 1 Orthopaedics Surgery and Sports Medicine Department, FIFA Medical Centre of Excellence, Croix-Rousse Hospital, Lyon University Hospital Lyon France; 2 LIBM – EA 7424, Interuniversity Laboratory of Biology of Mobility Claude Bernard Lyon 1 University Lyon France; 3 Univ Lyon, Claude Bernard Lyon 1 University, IFSTTAR, LBMC UMR_T9406 69622 Lyon France; 4 Department of Trauma and Orthopaedic Surgery, University College London Hospitals NHS Foundation Trust 235 Euston Rd. London NW1 2BU UK; 5 Division of Surgery and Interventional Science, University College London Gower Street London WC1E 6BT UK

**Keywords:** Covid-19, Total hip replacement, Outpatient surgery, Length of stay, Discharge destination, Rehabilitation

## Abstract

*Introduction*: The COVID-19 pandemic has significantly affected access to timely care for patients with hip osteoarthritis requiring total hip replacement (THR). This study aimed to assess the changes in surgical activity, outpatient treatment, length of stay (LOS), discharge destinations, readmission rates, clinical outcomes, and patient satisfaction before and after the pandemic at our institution. *Materials and methods*: This retrospective study encompassed patients undergoing primary THR through the direct anterior approach at a single university hospital. Data on demographic characteristics, surgical technique, perioperative management, LOS, discharge destinations, complications, and clinical outcomes were collected. Furthermore, a comparative analysis between the pre-pandemic (2019) and post-pandemic (2022) periods was conducted. *Results*: There was a 14% increase in surgical activity post-pandemic, with 214 patients undergoing surgery in 2019 versus 284 in 2022. The percentage of patients managed as outpatients significantly increased from 0.5% in 2019 to 29.6% in 2022 (*p* < 0.001). LOS decreased from 2.7 ± 1 [0–8] days to 1.4 ± 1.1 [0–12] days (*p* < 0.001), and the rate of discharge to rehabilitation centres declined from 21.5% to 8.8% (*p* < 0.001). No significant increase in the readmission rates was observed (1.4% in both periods). At two months postoperatively, the mean HHS and satisfaction rates were comparable between the two groups (*p* = 1 and *p* = 0.73, respectively). *Discussion*: Despite the challenges posed by the COVID-19 pandemic, surgical activity at our institution demonstrated an increase compared to the pre-pandemic levels by expanding outpatient care, reducing LOS, and increasing rates of home discharges. Importantly, these changes did not adversely affect rehospitalization rates or early clinical outcomes.

Level of evidence: IV

**Abbreviations**
THR: Total hip replacement
LOS: Length of stay
HHS: Harris hip score
DAA: direct anterior approach
BMI: body mass index
ASA: American Society of Anesthesiologists

## Introduction

Hip osteoarthritis has a profound impact on quality of life and mortality [1, 2]. Timely access to care, particularly surgical intervention, is therefore crucial for patients with advanced disease. However, during the recent COVID-19 pandemic, the number of patients with severe osteoarthritis waiting for total hip replacement (THR), enduring pain described as “worse than death”, nearly doubled [3]. This surge in cases can be attributed to the repeated postponement of non-urgent scheduled surgeries between March 2020 and December 2021 [4, 5]. Moreover, the challenges of restoring surgical activity to pre-pandemic levels have been exacerbated by healthcare staff shortages, limiting the utilization of operating theatres and hospital beds [6–8].

In response to these circumstances, our institution implemented a series of adaptations to ensure equitable access to THR. These institutional modifications primarily focused on reducing the average length of stay (LOS), expanding the utilisation of outpatient surgeries, and minimizing the need for rehabilitation centre admissions. There is mounting evidence suggesting the safety and efficacy of outpatient THR [9–12], with some groups reporting up to 75% utilisation of outpatient management [13]. While the transition to outpatient surgery typically involves a stepwise process amongst all stakeholders involved in patient care [14–16], the recent pandemic has necessitated rapid adaptation in practice.

The primary objective of this study was to assess the changes in the number of patients undergoing THR for hip osteoarthritis at our institution before (2019) and after (2022) the COVID-19 pandemic. Additionally, we aimed to investigate changes in the rate of outpatient treatment, LOS, and discharge destinations. Furthermore, we evaluated whether the swift adaptations to our practice had any impact on the rate of readmissions, clinical outcomes or patient satisfaction.

## Materials and methods

### Patients

This was a retrospective cohort study encompassing patients who underwent primary THR through the direct anterior approach (DAA) for hip osteoarthritis at a university hospital centre. All procedures were performed between 2019 and 2022. The preferred approach in our department was the Hueter anterior approach, while the posterior approach was utilised in specific cases such as anatomical abnormalities (surgical sequelae, fracture sequelae, major joint destruction, developmental dysplasia of the hip), obesity >40 kg/m^2^, or advanced age over 90 years old.

### Surgical technique

All patients included underwent THR using the Hueter Gaine approach, following the surgical technique described by Lustig [17] and Foissey et al. [18]. The procedure was conducted with the patient in the supine position, without the use of a traction table. Systematic fluoroscopic examination was conducted and no surgical drains were utilized. Dual mobility cups were used for patients older than 65 and those with a high risk of dislocation (e.g. epilepsy, Parkinson’s disease, substance abuse) [19, 20].

### Perioperative management

Patients scheduled to undergo inpatient THR were admitted on the morning of the surgery, with the option of admission the day before in cases of transportation difficulties or medical reasons upon the anaesthetist’s request. Patients scheduled to undergo outpatient THR were admitted on the morning of the surgery. Patients living alone at home, those with insufficient support or an ASA (American Society of Anesthesiologists) score ≥ 3 were not deemed eligible for ambulatory care. Age and BMI were not considered as exclusion criteria.

Spinal anaesthesia was the preferred type of anaesthesia. General anaesthesia was implemented if the patient declined or based on the anaesthetist’s judgment, considering factors such as spinal access or coagulation disorders. Following the surgery, patients were monitored in the recovery room and transferred back to their rooms once their Aldrete score exceeded 8 [21]. A fast-track postoperative recovery protocol was implemented, allowing patients to mobilize on the same day as the operation. Pain management involves a combination of level I to level III analgesics, non-steroidal anti-inflammatory drugs, and non-narcotic painkillers (Nefopam). Enoxaparin thromboprophylaxis was administered to all patients for 30 days.

Patients were discharged home once they met all the criteria outlined in Chung’s score [20]: satisfactory autonomy (ability to walk with canes for 70 m and climb stairs), restoration of normal urination, controlled pain (pain score <3/10 at rest, <5/10 with movement), clean incision site, absence of complications requiring medical supervision or additional surgery, and no nausea or drowsiness. If a patient’s background posed challenges for home discharge, admission to the rehabilitation centre was planned during the preoperative consultation.

During outpatient treatment, patients were discharged home with an intravenous line, and a nurse administered Nefopam in the morning, noon, and evening for two days. If level I and II analgesics and Nefopam were ineffective, patients were provided with a prescription for a level III analgesic. A follow-up phone call by a nurse was conducted the following day to ensure a smooth transition at home and the absence of any concerns.

### Data collection

Demographic data including age, sex and BMI, as well as activity level according to Devane et al. [22], ASA score, and Harris score (HHS) [23] were collected during the pre-operative consultation. The type of management (inpatient with admission the day before or the same day, outpatient), home discharge or discharge to a rehabilitation centre, early peri- and postoperative complications, and LOS were documented during the hospital stay. Outpatient management was considered to have a LOS equal to 0. Routine follow-up appointments were scheduled at 2 months, 1 year, and every 2–5 years thereafter. Herein, we report 2 months’ outcomes including HHS, satisfaction (disappointed, satisfied, very satisfied), complications and need for re-admission.

## Statistics

Continuous variables are presented as means with standard deviations. Statistical tests used included Student’s t-test for independent and normally distributed variables; paired Student’s *t*-test for paired, normally distributed variables; the non-parametric Mann-Whitney test for independent, non-normally distributed variables and the non-parametric Wilcoxon signed ranks test for paired, non-normally distributed variables. Categorical variables are presented as percentages and the Chi-squared or Fisher’s exact tests are used for comparisons. The significance level was set at 5%. The statistical analysis was performed using the XLstat software (version 2015.1, Addinsoft, France).

### Ethics approval

All procedures performed in studies involving human participants were in accordance with the ethical standards of the institutional and/or national research committee and with the 1964 Helsinki Declaration and its later amendments or comparable ethical standards. The Advisory Committee on Research Information Processing in the Field of Health (CCTIRS) approved this study on June 4, 2015, under number 15-430. For this type of study, formal consent is not required.

## Results

In 2019, a total of 214 patients underwent primary hip arthroplasty, excluding 76 patients operated on using the posterior approach, whereas in 2022, the number increased to 284 patients, excluding 69 patients operated on using the posterior approach, representing a 14% rise. No patients were lost to follow-up. Analyses of clinical data revealed no significant differences between the two patient populations, as shown in Table 1. Comparatively, patients managed as outpatients in 2022 were younger and had lower ASA scores compared to patients managed as inpatients (Table 2).

**Table 1:** Demographics, pre-and post-operative clinical data and early rehospitalisation of patients operated on in 2019 and 2022.

	2019 (*n* = 214)	2022 (*n* = 284)	*p*-Value
Gender			0.49
Women	113 (52.8%)	141 (49.6%)	
Men	101 (47.2%)	143 (51.4%)	
Age (years)	66.4 ± 9.1 [12.4–87.6]	68.1 ± 8.1 [12.1–88.2]	0.17
BMI (kg/cm)^2^	25.7 ± 3.3 [15.6–38.9]	26 ± 3.2 [17.5–38.9]	0.89
Etiology			0.20
Primary osteoarthritis	188 (87.9%)	264 (93%)	
Osteonecrosis	16 (7.5%)	12 (4.2%)	
Post-traumatic	3 (1.4%)	4 (1.4%)	
Dysplasia	7 (3.2%)	4 (1.4%)	
Pre-operative HHS (/100)	61.3 ± 2.5 [44–68.5]	59.4 ± 2.3 [45.5–64.5]	0.65
ASA score	1.9 ± 0.5 [1–4]	1.9 ± 0.4 [1–4]	0.97
Devane activity score	3.6 ± 0.6 [2–5]	3.7 ± 0.5 [2–5]	0.82
Type of anaesthesia			<0.001
General	24 (11%)	63 (22%)	
Spinal	190 (89%)	221 (78%)	
LOS (Days)	2.7 ± 1 [0–8]	1.4 ± 1.1 [0–12]	<0.001
Rehospitalisation < 2 months	3 (1.4%)	4 (1.4%)	1
Aetiology of rehospitalisation			1
Acute sepsis	1 (0.5%)	1 (0.4%)	
Haematoma	1 (0.5%)	1 (0.4%)	
Need for transfusion	0	1 (0.4%)	
Fall	0	1 (0.4%)	
Pulmonary embolism	1 (0.5%)	0	
HHS 2 months (/100)	82.3 ± 1.9 [78–90]	82.3 ± 1.5 [70–91]	1
Satisfaction at 2 months			0.73
Very satisfied	181 (84.6%)	247 (87%)	
Satisfied	30 (14%)	33 (11.6%)	
Disappointed	3 (1.4%)	4 (1.4%)	

BMI: body mass index, HHS: Harris Hip Score, ASA: American Society of Anesthesiologists, LOS: average length of stay.

**Table 2:** Comparison of patients managed in an outpatient or inpatient setting in 2022.

	Outpatient (*n* = 84)	Inpatient (*n* = 200)	*p*-Value
Gender			0.94
Women	42 (50%)	99 (49.5%)	
Men	42 (50%)	101 (50.5%)	
Age (years)	65.1 ± 7.8 [32.0–81.5]	69.4 ± 8.2 [12.0–88.0]	<0.005
BMI (kg/cm)^2^	26.1 ± 2.9 [18.7–37.1]	26.0 ± 3.4 [17.5–38.9]	0.92
Etiology			0.62
Primary osteoarthritis	77 (91.7%)	187 (93.5%)	
Osteonecrosis	3 (3.5%)	9 (4.5%)	
Post-traumatic	2 (2.4%)	2 (1%)	
Dysplasia	2 (2.4%)	2 (1%)	
Pre-operative HHS (/100)	59.3 ± 2.2 [45.5–64.0]	59.4 ± 2.3 [50.0–64.5]	0.84
ASA score	1.6 ± 0.5 [1.0–3.0]	2.0 ± 0.4 [1.0–4.0]	<0.005
Devane activity score	3.6 ± 0.5 [2.0–5.0]	3.7 ± 0.5 [2.0–5.0]	0.73
DMS (joys)	0	2.0 ± 1.1 [1.0–12.0]	<0.005
Rehospitalisation < 2 months	1 (1.2%)	3 (1.5%)	1
Aetiology of rehospitalisation			1
Acute sepsis	1 (1.2%)	0	
Haematoma	0	1 (0.5%)	
Transfusion	0	1 (0.5%)	
Fall	0	1 (0.5%)	
Pulmonary embolism			
HHS 2 months (/100)	82.7 ± 1.4 [78.8–90.0]	82.2 ± 1.6 [70.0–90.6]	0.68
Satisfaction 2 months			0.41
Very satisfied	76 (90.5%)	171 (85.5%)	
Satisfied	8 (9.5%)	25 (12.5%)	
Disappointed	0	4 (2%)	

BMI: body mass index, HHS: Harris Hip Score, ASA: American Society of Anesthesiologists, LOS: average length of stay.

During the period spanning 2019 to 2022, there was a notable increase in the rate of patients undergoing outpatient surgery, with frequencies of 0.5% (1/214) in 2019 and 29.6% (84/284) in 2022, yielding a statistically significant difference (*p* < 0.001). In 2019, there were no cases of outpatient management failures. In 2022 outpatient management failed for a total of five patients (5.6%, 5/89), with one case of uncontrolled pain, two cases of acute urinary retention, and two cases of malaise.

The LOS decreased significantly (2.7 ± 1 [0–8] days versus 1.4 ± 1.1 [0–12] days, *p* < 0.001). This decrease was manifested even when excluding patients managed on an outpatient basis (2.7 ± 1 [1–8] days versus 2 ± 1 [1–12] *p* < 0.001). Additionally, the rate of discharge to rehabilitation centres decreased from 21.5% (46/214) in 2019 to 8.8% (25/284) in 2022, showing a significant decline (*p* < 0.001).

No noticeable increase was observed in the rate of readmissions; 1.4% (3/214) in 2019 versus 1.4% (4/284) in 2022, *p* = 1). Detailed aetiology of rehospitalisation can be found in Table 1. Furthermore, aside from the reported causes of readmission, no other early complications were comparable to HHS (82.3 ± 1.9 [78–90] in 2019 versus 82.3 ± 1.5 in 2022 [70–91], *p* = 1). Similarly, no statistically significant differences were noted in the satisfaction rate (84.6% very satisfied (181/214), 14% satisfied (30/214), 1.4% disappointed (3/214) versus 87% very satisfied (247/284), 11.6% satisfied (33/284), 1.4% disappointed (4/284), *p* = .73).

Patients undergoing outpatient THR in 2022 exhibited comparable rehospitalisation rates, HHS scores and satisfaction compared to inpatient THR (Table 2).

## Discussion

Despite the challenges posed by the COVID-19 pandemic, our hospital successfully achieved a 14% increase in the provision of surgeries for patients with hip osteoarthritis between 2019 and 2022. This rise in surgical activity was facilitated by two key factors: a notable expansion of outpatient care, which accounted for 30% of patients in 2022, and a reduction of 1.3 days in LOS.

Several registry studies have reported a significant decline in elective joint replacement surgeries in 2020 due to the Covid-19 pandemic. Specifically, a decrease of 20% in the Netherlands, 5% in Denmark, 47% in the United States, and 45% in Great Britain has been indicated [24–26]. Consequently, this has resulted in longer waiting times and an unacceptable health state of the patients [3]. In 2021, Oussedik et al. estimated that, even with a 30% increase in productivity, it would take between 20 and 48 months to restore waiting lists to pre-pandemic levels in Great Britain [26]. While numerous studies have focused on the reduction in orthopaedic activity during the pandemic, only a few have provided post-pandemic figures. Our series demonstrates a 14% increase in surgical activity between the pre- and post-pandemic periods, driven by the growing demand from patients.

While the shift towards ambulatory surgery is often stepwise [14–16], our institution faced a swift change in practice. Mitchell et al. recently disseminated findings of a similar study, comparing the management evolution between 2019 and 2020 at a single centre in the United States [27]. They have profoundly changed their overall approach to patient care, for example by prioritising earlier postoperative return to function by increasing the use of spinal anaesthesia over general anaesthesia, reducing the dose of bupivacaine in spinal anaesthesia and eliminating intrathecal narcotics. Their results are in concordance with our study, indicating an increase in outpatient care from 0.1% to 28.9% and a reduction in the mean LOS from 1.3 to 0.9 days. This trend has been observed nationwide in the United States, where a national analysis demonstrated a rise in outpatient care from 5.7% to 35.6% [28]. These encouraging results could support the notion of the eligibility criteria for routine outpatient surgery, allowing for the inclusion of older patients with more comorbidities, as was implemented during the COVID-19 pandemic [29].

The readmission rate of 1.4% in our study closely aligns with the rate of 1.5% reported by Bemelmans et al. in a recent meta-analysis of patients managed solely on an outpatient basis [30]. Furthermore, evidence suggests that outpatient management may have a protective effect against readmissions and complications when appropriate patient selection and eligibility criteria are applied. Based on these reassuring results, several teams advocate for extending the conventional criteria for outpatient THR, particularly for patients with an ASA score ≥3 [13, 31].

Our study has several limitations that warrant acknowledgement. Firstly, we standardised our surgical approach encompassing patients who underwent primary THR via the direct anterior approach to ensure homogeneity and avoid introducing confounders by including surgeries performed via the posterior approach. Hence, it should be acknowledged that the overall rate of outpatient surgery in our department in 2022 (24%, 84/353) is lower than reported in this study. Secondly, with respect to Patient Reported Outcome Scores (PROMs), our current practice solely incorporates the collection of HHS, which could limit the adjudication of functional results. Thirdly, our study design could have potentially introduced selection bias, owing to its retrospective nature. Finally, the unique impact of the COVID-19 pandemic on individual hospitals and the significant variation in the organizational response could potentially impact the generalisability of our findings.

## Conclusions

Our findings demonstrated that despite the challenges faced in the post-COVID era, our institutional provision of primary THA demonstrated a 14% increase. The key pillars of this accomplishment include the expansion of outpatient care capacity, a reduction in the LOS, and increased rates of home discharges. Importantly, these changes have not adversely affected rehospitalization rates or early clinical outcomes.

## Conflicts of interest

C.F: Arthrex hospitality benefit unrelated to the article.

T.P: No conflicts of interest.

E.S.: Consultant for Corin, not related to the article.

AF: Onassis Foundation Scholarship, Freemasons’ Royal Arch Fellowship with support from the Arthritis Research Trust all unrelated to this work.

C.B.: Stryker hospitality advantage, Groupe Lépine sponsorship, not related to the article.

S.L.: Consultant for Stryker, Smith Nephew, Heraeus, Depuy Synthes, Institutional research support from Groupe Lepine, Amplitude unrelated to the article.

## Funding

The authors received no specific funding for this work.

## Authors’ contributions

CF: data acquisition, analysis and interpretation, writing. TP: data acquisition. ES: critical revision. AF: critical revision CB: surgical procedures, critical revision. SL: critical revision.
